# Metabolome and Transcriptome Analyses Reveal Metabolomic Variations and Key Transcription Factors Involved in Lipid Biosynthesis During Seed Development in *Carya illinoinensis*

**DOI:** 10.3390/ijms252111571

**Published:** 2024-10-28

**Authors:** Kaikai Zhu, Lu Wei, Hammad Hussain, Pengpeng Tan, Guo Wei, Juan Zhao, Sichen Zhou, Hui Liu, Fangren Peng

**Affiliations:** 1 Co-Innovation Center for Sustainable Forestry in Southern China, Nanjing Forestry University, Nanjing 210037, China; kkzhu@njfu.edu.cn (K.Z.); wl18236975076@163.com (L.W.); tanpengpeng2002@163.com (P.T.); cxyl@njfu.edu.cn (J.Z.); zzsichen@126.com (S.Z.); 2 College of Horticulture and Landscape Architecture, Yangzhou University, Yangzhou 225009, China; hussainhammad788@gmail.com (H.H.); gwei@yzu.edu.cn (G.W.); 3 State Key Laboratory of Crop Genetics and Germplasm Enhancement, Ministry of Agriculture and Rural Affairs Key Laboratory of Biology and Germplasm Enhancement of Horticultural Crops in East China, College of Horticulture, Nanjing Agricultural University, Nanjing 210095, China; liuhui@njau.edu.cn

**Keywords:** *Carya illinoinensis*, expression patterns, metabolite, lipid biosynthesis, transcriptome

## Abstract

Plant oils are a large group of neutral lipids that play a vital role in the food and oleochemical industries. The pecan (*Carya illinoinensis*) is a promising woody oil crop known for its high-quality sources of essential fatty acids and various bioactive compounds that may aid in preventing heart diseases. However, there is still a lack of understanding regarding the accumulation of lipids and the molecular mechanism of lipid biosynthesis during seed development. This study aims to analyze the metabolite variations and molecular mechanisms of lipid biosynthesis by integrating untargeted metabolomics and transcriptomics during pecan seed development. A total of 293 differentially accumulated metabolites were identified and further categorized into 13 groups, with lipids and lipid-like molecules constituting the largest group. The oil content and fatty acid compositions of pecan embryos were assessed at three stages of seed development. Oleic acid (c18:1) and linoleic acid (c18:2n6) were found to be the most abundant unsaturated fatty acid components in pecan embryos. Additionally, a comprehensive analysis revealed 15,990 differentially expressed genes, with a focus on the key genes related to lipid metabolism. Furthermore, the study identified 1201 transcription factors from differentially expressed genes. These transcription factors were divided into 65 families, with different members in the same family exhibiting different expression patterns during seed development. The expression patterns of ten transcription factor genes during seed development were verified by qRT–PCR. Two key genes, *CiABI3* and *CiFUS3* were further cloned and found to be localized in the nucleus. This study used metabolome and transcriptome analysis during key periods of pecan seed development to identify the key genes associated with seed development and fatty acid biosynthesis.

## 1. Introduction

The pecan tree (*Carya illinoinensis*) is well known as a nut-bearing species in the *Carya* family, renowned for its delicious nuts and high nutritional value [1]. This tree species is native to the United States and Mexico, and pecans boast a kernel oil content exceeding 70%, with unsaturated fatty acids comprising over 90% and relatively low saturated fatty acids [2]. These unsaturated fatty acids offer various health benefits, including reducing blood lipids and preventing cardiovascular diseases. Moreover, pecan nuts are rich sources of essential minerals, such as magnesium and phosphorus, which play a crucial role in overall health and metabolic functions [3]. Plant oils are stored primarily as triacylglycerols (TAGs) in plants, serving as carbon sources and energy reservoirs [4]. The metabolic pathways of plant lipids are complex, involving a cascade of enzymatic reactions and transcriptional regulation across various organelles [5]. Fatty acid synthesis, catalyzed by fatty acid synthase complexes in plastids, is a central process in oil biosynthesis. Acetyl-CoA, an important substrate for fatty acid synthesis, is catalyzed by acetyl-CoA carboxylase (ACCase) to form malonyl-CoA, initiating fatty acid elongation [6].

Plant seed oils are influenced by a variety of functional genes, including diacylglycerol acyltransferase (DGAT), ketoacyl coenzyme A synthetase (KCS), stearoyl ACP desaturase (SAD), and fatty acid desaturase (FAD), which play key roles in the composition and accumulation of fatty acids [5]. Transcription factors (TFs) are a type of DNA-binding proteins, which positively or negatively regulate the expression of target genes by interacting with specific DNA sequences in the promoter regions. They are involved in various biological processes, such as development and abiotic/biotic stress response, including lipid biosynthesis in plants [7]. TFs, such as FUSCA3 (FUS3), ABSCISIC ACID INSENSITIVE3 (ABI3), LEAFY COTYLEDON1 (LEC1), and LEAFY COTYLEDON2 (LEC2) play a crucial role in regulating plant oil biosynthesis during seed development. These four TFs, collectively referred to as LAFL (LEC1, ABI3, FUS3, and LEC2), influenced the expression of the genes involved in glycolysis and fatty acid synthesis pathways through the downstream regulator WRINKLED1 (WRI1), a member of the AP2-EREBP transcription factor family [8]. The overexpression of the WRI1 gene has been shown to increase seed oil in various plant species, such as *Arabidopsis*, rapeseed, maize, and flax, emphasizing its pivotal role in lipid metabolism [9].

Recently, integrated omics approaches have been utilized to combine transcriptomics, genomics, metabolomics, and proteomics analyses for basic research and plant breeding. These approaches have been applied to study plant growth, development, and response to various stresses, providing a comprehensive understanding of these processes [10,11]. The accumulation of oil in embryos is a major feature during seed development in the walnut and the pecan [2,12]. A combination of metabolome and transcriptome analysis was utilized to compare the kernel lipid composition of walnuts and identify the key genes in the lipid metabolism pathway in the walnut [13]. Previous studies have shown that the key genes involved in lipid metabolism were identified in the pecan through transcriptome analysis, highlighting a series of enzymes involved in the de novo biosynthesis of fatty acids and the synthesis of TAG [1,2,14,15]. In recent years, metabolomics has emerged as a useful tool to examine the metabolic changes associated with plant growth and development. Zhang et al. combined metabolomics and transcriptomics analyses to explore variations in the contents of bioactive components throughout pecan kernel development [16]. Fifty-eight lipid types that are mainly composed of glycerolipids and phospholipids were found according to lipidomic analyses of the pecan kernel [15]. Recently, integrated lipidomic and transcriptomic analysis were used to find the key genes involved in pecan oil biosynthesis, and predicted a group of TFs involved in the regulation of fatty acid biosynthesis [17]. In this study, we integrated transcriptomics and untargeted metabolomics to investigate variations in the contents of components and their molecular pathways during pecan seed development. Our findings may aid in elucidating the key regulatory networks involved in lipid biosynthesis during pecan seed development and identify key regulatory genes and networks that can improve the quality of pecan nuts and increase oil yield.

## 2. Results

### 2.1. Morphological and Oil Content Analysis of Pecan Developing Embryo Samples

**Figure 1:**
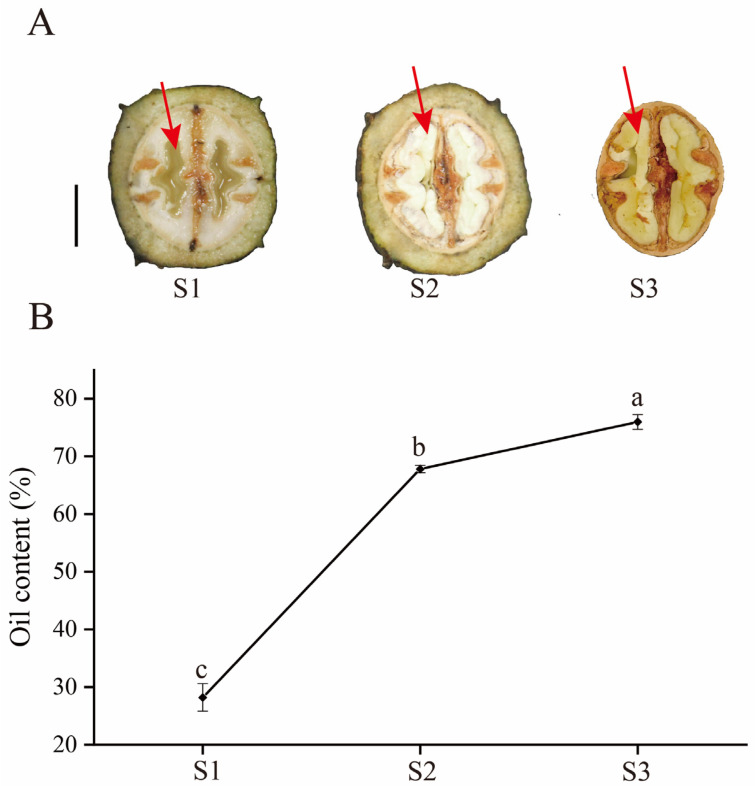
Morphological characteristics and oil content changes in the process of pecan embryo development. (**A**) Phenotypic variation of pecan seeds during three different development stages. Bar = 1 cm. Red arrows indicate samples we collected. (**B**) Oil contents of pecan embryo samples during three stages of seed development. The data are mean value ± SE of three replicates, and bars with different letters represent statistically significant at *p* < 0.05 by Duncan’s multiple range test.

Pecan seeds at three harvest stages, including stage 1 (S1), stage 2 (S2), and stage 3 (S3), were selected to determine the physiological changes during seed maturation; we found the embryo samples changed from watery to creamy yellow (Figure 1A). The oil content analysis revealed a continuous accumulation of oil throughout pecan seed development (Figure 1B). Notably, the oil content in the embryos at S2 was 140.4% higher than that at S1, indicating a rapid increase between these stages. However, by S3, the oil content showed only a 12.1% increase compared to S2, indicating a slight increase during the later stage of development. Our results revealed that oil accumulation in the pecan is an important feature from S1 to S3.

### 2.2. Metabolic Differences in Embryo Samples During Pecan Seed Development

Based on the morphological and oil content analysis, an LC-MS-based metabolomics approach was further applied to investigate the dynamic changes of metabolites during pecan seed development. A total of 41,647 substances were identified in both positive (22,654) and negative (18,993) ion models through untargeted metabolomics analysis (Table S1). According to the annotated substances using secondary MS (MS2), 672 metabolites in positive ion mode (POS) and 268 in negative ion mode (NEG) with reliable annotations were selected for further analysis (Table S2).

**Figure 2:**
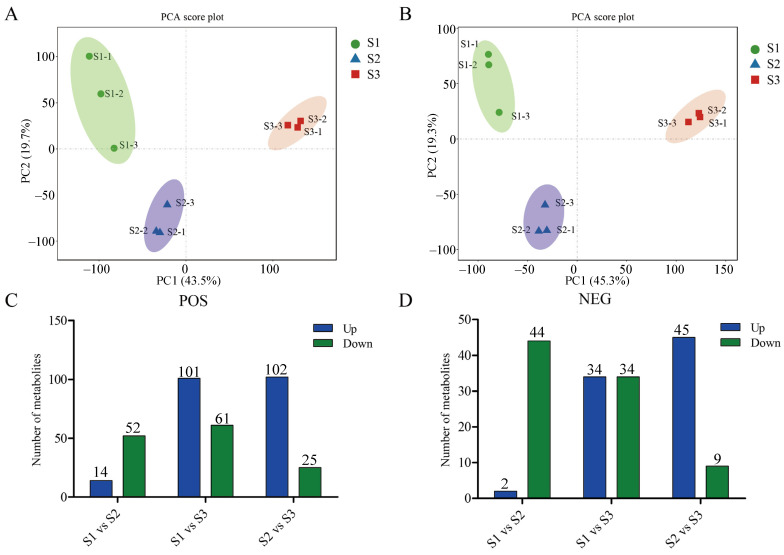
Differentially accumulated analysis of metabolites during pecan seed development. (**A**) PCA score plots of metabolic profiles from all samples generated by the POS ion model. (**B**) PCA score plots of metabolic profiles from all samples generated by the NEG ion model. (**C**) Number of DAMs among the different comparison sets by the POS model. (**D**) Number of DAMs among the different comparison sets by the NEG model.

Principal component analysis (PCA) was performed on all nine samples in both the positive (Figure 2A) and negative (Figure 2B) ion models. The PCA results demonstrated that the embryo samples from different developmental stages were categorized into distinct groups, suggesting a clear difference in the metabolite profiles among the various stages.

Furthermore, we have identified a total of 293 differentially accumulated metabolites (DAMs) across all comparison sets (Table S3). In the POS mode, 197 DAMs were identified. In the S1 (stage 1) vs. S2 (stage 2) set, 66 DAMs were found, with 14 up-regulated and 52 down-regulated. In the S1 vs. S3 (stage 3) set, 162 DAMs were identified, with 101 up-regulated and 61 down-regulated. The S2 vs. S3 set revealed 127 DAMs, with 102 of them being up-regulated and 25 being down-regulated (Figure 2C). Moreover, in the NEG mode, 96 DAMs were identified. Specifically, 46 DAMs (2 up-regulated and 44 down-regulated) were discovered in the S1 vs. S2 set, and 68 and 54 DAMs were identified in the S1 vs. S3 and S2 vs. S3 sets, respectively (Figure 2D).

**Figure 3:**
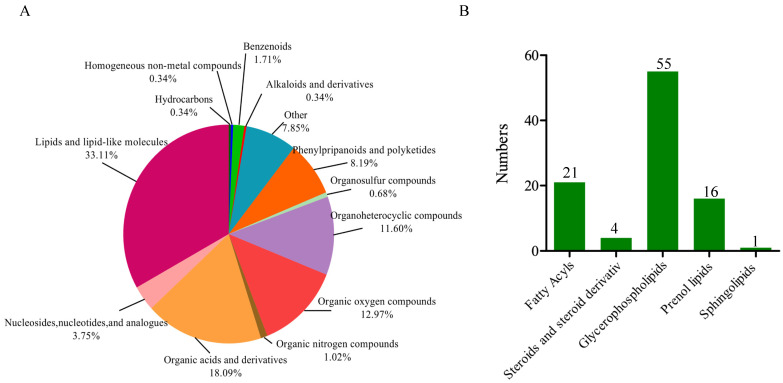
Classification of differentially accumulated metabolites in pecan embryo during seed development. (**A**) Classification of the DAMs into groups. (**B**) Subgroups involved in the lipids and lipid-like molecules group.

We further classified the 293 DAMs into 13 groups, with lipids and lipid-like molecules being the largest group (33.11%), which contained 97 DAMs. This indicated that lipid biosynthesis played an important role during the three seed developmental stages (Figure 3A). In the lipids and lipid-like molecules group, a total of 97 DAMs were categorized into five subgroups. The two largest subgroups were glycerophospholipids and fatty acyls, containing 55 and 21 DAMs, respectively (Figure 3B). The expression patterns of the 97 DAMs revealed that more than half of the DAMs peaked at S3, indicating that they accumulated in the late stage of embryo development (Figure S1). This detailed metabolomic analysis highlighted the dynamic nature of metabolite accumulation during pecan seed development, providing valuable insights into the metabolic pathways involved in seed maturation and lipid biosynthesis. KEGG pathway enrichment, with the top 20 significantly enriched pathways across different comparisons, was investigated (Figure S2), and we found fatty acid metabolism and fatty acid degradation were present in the S1 vs. S3 comparison. Interestingly, DAMs were enriched in the biosynthesis of plant hormones across all three comparisons, indicating that plant hormones participated in the seed-development process of the pecan.

### 2.3. Lipid Composition Analyses in Pecan Embryos During Seed Development

In this study, the fatty acid compositions of pecan embryos were evaluated at three stages of seed development. A total of 17 FAs were detected across these stages, including 7 types of saturated FAs and 10 types of unsaturated FAs (Table 1). Notably, most FA components exhibited continuous increases from S1 to S3. Among the seven saturated FAs, palmitic acid (c16:0) and stearic acid (c18:0) were the most abundant components, with their contents increasing by 5.7 and 11.1 fold at S3 compared to S1, respectively. Additionally, pentadecanoic acid (C15:0) and tricosylic acid (C23:0) showed significant up-regulation during the early stages of seed development.

In pecan embryos, oleic acid (c18:1) and linoleic acid (c18:2n6) were the most abundant unsaturated FAs. The oleic acid content increased by 10.5 fold from S1 to S2, indicating a rapid accumulation during the early maturation stage of pecan seeds. Linoleic acid also showed significant accumulation during seed development.

### 2.4. Transcriptome Analysis of Pecan Seed Development

To investigate the changes in gene expression during the development of pecan seeds and the process of lipid biosynthesis, we collected embryo samples at three different developmental stages for transcriptome analysis and constructed a total of nine cDNA libraries. This resulted in 68.07 Gb of raw sequencing data, with the raw reads per library ranging from 45,622,914 to 62,026,706. After removing the low-quality reads, clean reads per library ranged from 45,500,322 to 61,864,952, with the proportion of clean reads exceeding 99% in all nine libraries. The Q30 values ranged from 93.91% to 94.22%, indicating high-quality sequencing results.

Subsequently, the sequencing data of each sample were mapped to the reference genome of the pecan. The mapping rate for each library ranged from 95.26% to 96.52% (Table S4). Over 88% of the aligned sequences were located in exon regions, while intron and intergenic regions accounted for 5.19% and 5.78%, respectively (Figure S3).

**Figure 4:**
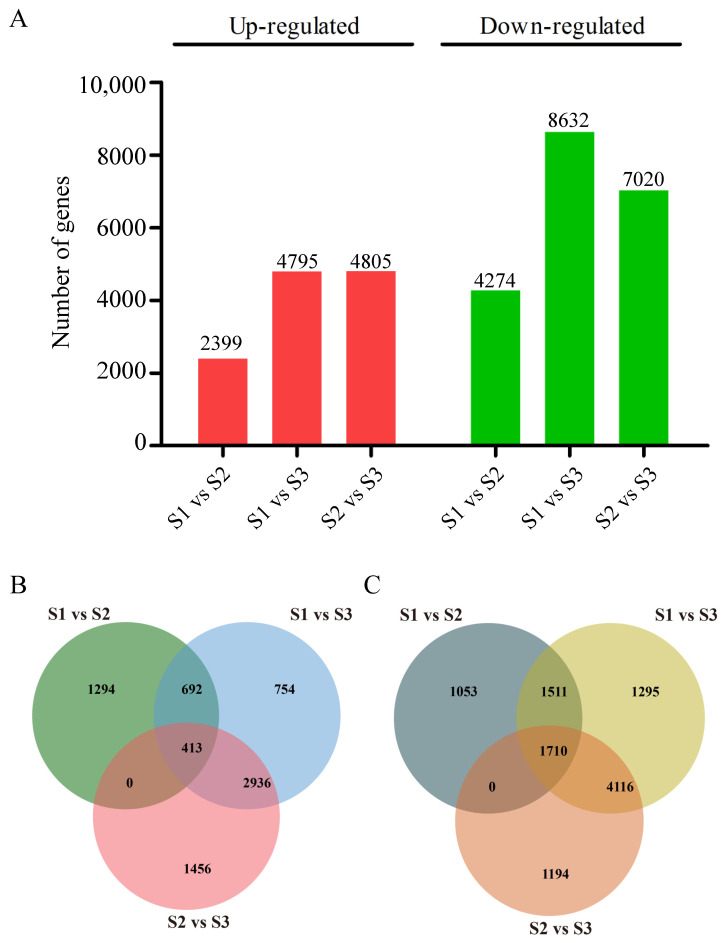
Differentially expressed genes in three developmental stages of pecan. (**A**) Number of the DEGs. (**B**) Venn diagram of overlapping and specific up-regulated DEGs. (**C**) Venn diagram of overlapping and specific down-regulated DEGs.

To identify the differentially expressed genes (DEGs) during pecan seed development, we compared the transcriptome data at the S1, S2, and S3 stages. A total of 15,990 DEGs were identified across all comparisons. In the S1 vs. S2 set, we found 6673 DEGs, with 2399 up-regulated and 4274 down-regulated genes. The S1 vs. S3 set revealed 13,427 DEGs, including 4795 up-regulated and 8632 down-regulated genes. In the S2 vs. S3 set, 11,825 DEGs were identified, with 4805 up-regulated and 7020 down-regulated genes (Figure 4A). A Venn diagram analysis of the DEGs indicated that 413 genes were consistently up-regulated across all three stages (Figure 4B), while 1710 genes were consistently down-regulated (Figure 4C). This suggests that these genes may play roles in pecan seed development and oil biosynthesis through their differential expression patterns. A KEGG enrichment analysis of the DEGs among the three development stages of pecan seeds showed that most DEGs were enriched with metabolic and biosynthesis of secondary metabolites (Figure S4).

### 2.5. Gene Expression and Metabolite Accumulation for Lipid Metabolism Pathway

**Figure 5:**
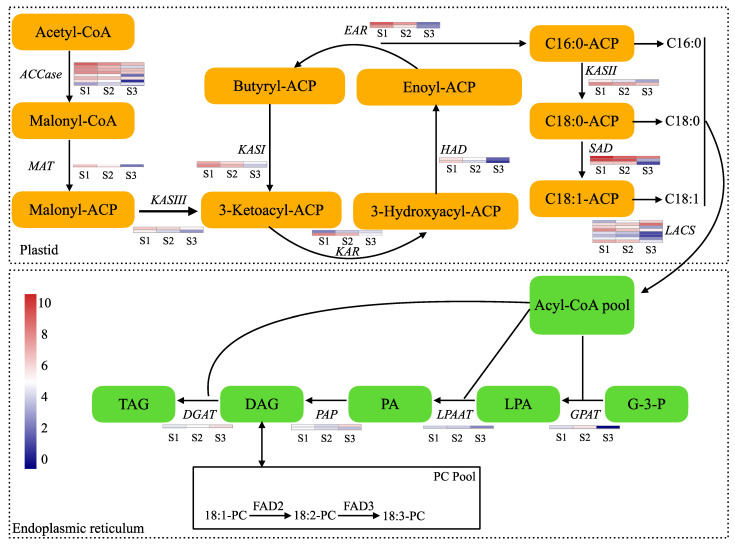
Fatty acid metabolism pathway map for pecan. The model diagram of the main fatty acid metabolism pathway and expression analysis of related genes during the development of pecan embryo. More information about the heatmap is provided in Figure S5. Accase, acetyl-CoA carboxylase; MAT, malonyl-CoA: ACP transacylase; KAS III, 3-ketoacyl synthase III; KAS I, 3-ketoacyl synthase I; KAR, 3-ketoacyl-ACP reductase; HAD, hydroxyacyl-ACP dehydratase; EAR, enoyl-ACP reductase; KAS II, 3-ketoacyl synthase II; SAD, stearoyl ACP desaturase; LACS, long-chain acyl-CoA synthetase; GPAT, glycerol-3-phosphate acyltransferase; LPAAT, 1-acyl-sn-glycerol-3-phosphate acyltransferase; PAP, phosphatidate phosphatase; DGAT, diacylglycerol acyltransferase; FAD2, oleoyl desaturase; FAD3, linoleoyl desaturase. G-3-P, glycerol-3-phosphate; TAG, triacylglycerol; LPA, lysophosphatidic acid; PA, phosphatidic acid; DAG, diacylglycerol; PC, phosphatidylcholine.

A comprehensive analysis of the key genes associated with lipid metabolism was carried out using the genomic data from the KEGG database to investigate the lipid metabolism pathway. Then, a model diagram of the main lipid metabolism pathway was constructed (Figure 5). The analysis revealed a set of 37 differentially expressed genes that potentially play a key role in pecan lipid metabolism, after filtering the low-expression-level genes. The initial step of fatty acid biosynthesis in the plastid is catalyzed by ACCase, with eight differentially expressed ACCase genes identified, seven of which were down-regulated from S1 to S3 (Table S5). Interestingly, most of the genes in the plastid, including the 1 *MAT* (*malonyl-CoA:ACP transacylase*), 2 *EAR* (*enoyl-ACP reductase*), 2 *HAD* (*hydroxyacyl-ACP dehydratase*), 1 *KAR* (*3-ketoacyl-ACP reductase*), 2 *KASI* (*3-ketoacyl synthase I*), 2 *KASII* (*3-ketoacyl synthase II*), 2 *KASIII* (*3-ketoacyl synthase III*), 3 *SAD,* and 6 *LACS* (*long-chain acyl-CoA synthetase*) genes, were significantly down-regulated. Meanwhile, in the endoplasmic reticulum, the *DGAT* (*diacylglycerol acyltransferase*), *PAP* (*phosphatidate phosphatase*), *LPAAT* (*1-acyl-sn-glycerol-3-phosphate acyltransferase*), and *GPAT* (*glycerol-3-phosphate acyltransferase*) genes exhibited various expression patterns.

### 2.6. Expression Patterns of Differentially Expressed Transcription Factors During Pecan Seed Development

**Figure 6:**
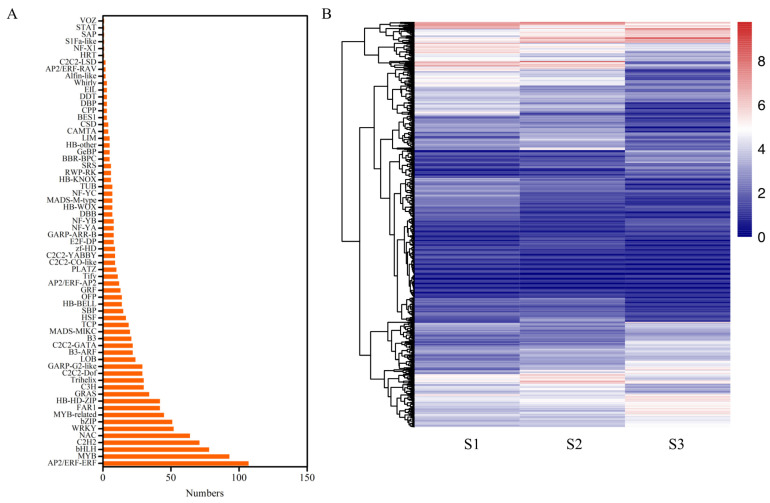
Differentially expressed transcription factors in three developmental stages of pecan seeds. (**A**) Classification of differentially expressed TF families. (**B**) Expression patterns of differential TFs during seed development. Log2 (FPKM+1) values were applied with the red–white–blue color scale. More details of expression data are listed in Table S6.

Transcription factors (TFs) are critical regulatory proteins that specifically bind to the cis-acting elements of target genes to modulate the expression of target genes. The plant TFs comprise hundreds of different families with distinct DNA-binding domains and functional characteristics. These TF families play specific roles in regulating plant growth, development, and environmental adaptation. According to RNA-Seq analysis, this study identified 1201 TFs from the 15,990 differentially expressed genes (DEGs). These TFs were classified into 65 families based on their structural domain sequences (Figure 6A). Among these TF families, six TF families contained only one gene, while seven families had more than 50 differentially expressed genes, including ERF (107), MYB (93), bHLH (78), C2H2 (71), NAC (64), WRKY (52), and bZIP (51), indicating their potential roles in pecan seed development.

The analysis of the expression patterns of these TF genes during pecan seed development revealed diverse expression profiles (Figure 6B). Around one-third of the TFs exhibited low expression levels. Notably, the majority of TF genes showed significant differential expression at the S3 stage. Furthermore, different members within the same family, such as the ERF family, demonstrated diverse expression patterns.

**Figure 7:**
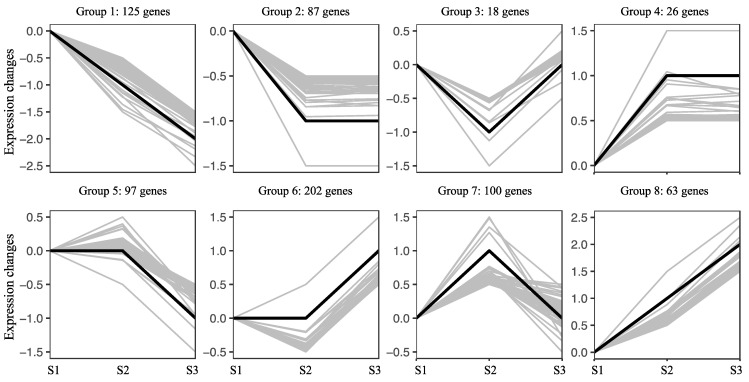
Expression trends of differentially expressed TFs in three developmental stages of pecan seeds. The number of TFs in each group is listed at the top of each group.

The preliminary differential gene-expression analysis identified a total of 1201 differentially expressed genes (DEGs). Low-expressing genes were filtered out to investigate the distinct expression trends of these transcription factors. Subsequently, the 718 genes were categorized into eight clusters (Table S7) based on their expression profiles during seed development (Figure 7). The analysis revealed that the largest group comprised 202 genes (Group 6). Meanwhile, 125 genes (Group 1) demonstrated continuous down-regulation from S1 to S3. Additionally, 97 genes (Group 5) showed no change from S1 to S2, followed by significant down-regulation from S2 to S3. Conversely, 63 genes (Group 8) exhibited consistent up-regulation from S1 to S3. The number of continuously down-regulated genes was approximately double that of continuously up-regulated ones (Table S8).

### 2.7. Expression Validation and Correlation Analysis of TF Genes and Subcellular Localization of CiABI3 and CiFUS3

**Figure 8:**
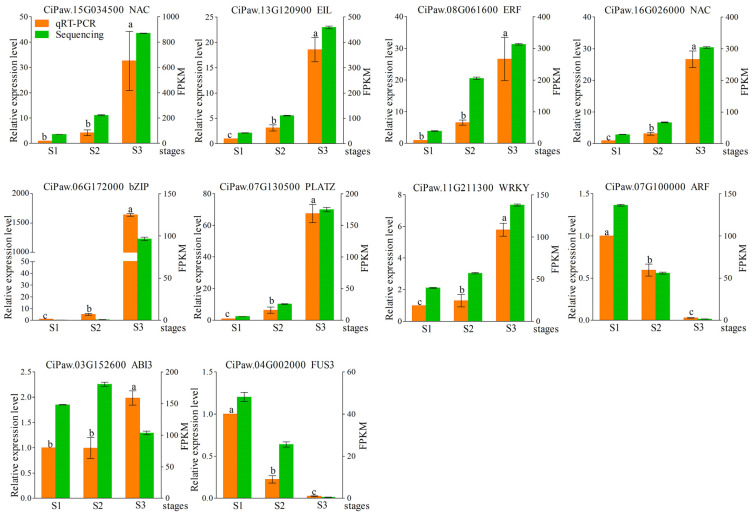
qRT–PCR validation of different TF genes during three seed developmental stages in pecan. The relative expression values of qRT–PCR (orange column) were referenced on the left *Y*-axis, and the FPKM value of RNA sequencing (green column) was referenced on the right *Y*-axis. The data are mean value ± SE of three replicates, and bars with different letters represent statistically significant at *p* < 0.05 by Duncan’s multiple range test. Primers are listed in Table S9.

To validate the accuracy of the transcriptome data, we selected 10 genes from various transcription factor families that were either up-regulated or down-regulated for qRT–PCR verification (Figure 8). The expression patterns of most of these genes were consistent with the transcriptome data, except *CiPaw.03G152600*, which showed different expression patterns between the qRT–PCR and the sequencing results. Particularly, eight genes were significantly up-regulated during seed development, with their highest expression levels observed at the S3 stage. Six genes, excluding *CiPaw.11G211300* and *CiPaw.03G152600*, showed more than a ten-fold increase in expression at S3 compared to S1 (Figure 8). Conversely, the expression levels of *CiPaw.07G100000* and *CiPaw.04G002000* gradually decreased throughout the seed-development process.

A correlation analysis was performed to investigate the mutual relationships between the 10 selected TF genes and the 37 genes related to fatty acid metabolism (Table S5). The networks consisted of 47 nodes (genes) and 448 edges, forming one main network and one subnetwork (Figure S6). The edges of the TF genes ranged from 2 to 28, with *CiFUS3* containing the maximum number of edges, indicating its central role in lipid metabolism.

**Figure 9:**
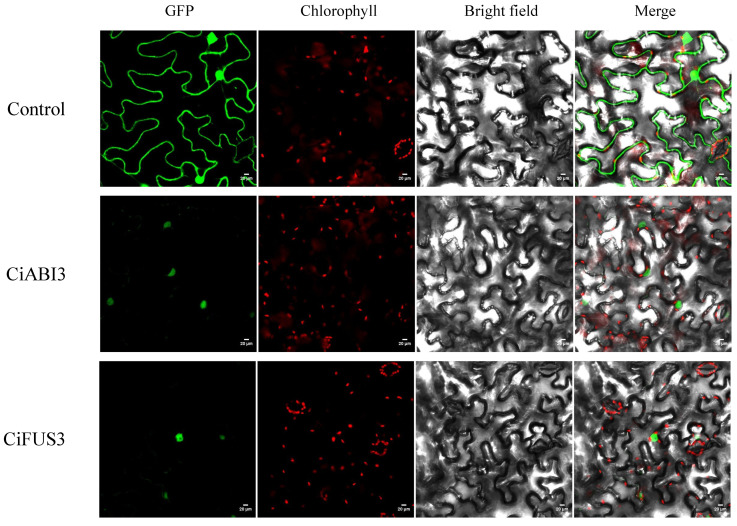
Subcellular localization of CiABI3 and CiFUS3 by the transient expression of a fused fluorescent protein in tobacco leaves. The two TF genes were cloned from pecan embryo samples and used to construct CaMV35S::CiABI3-GFP and CaMV35S::CiFUS3-GFP vectors. Scale bar = 20 μm. Control, GFP alone. Primers were listed in Table S10.

The B3 transcription factor family members (ABI3 and FUS3) are known to play a crucial role in plant oil biosynthesis. The subcellular localization of proteins can reveal their potential functions. Based on the gene-expression data, the *CiABI3* and *CiFUS3* genes were successfully cloned and subsequently fused with the green fluorescent protein (GFP) reporter gene. The recombinant vectors were transiently expressed in tobacco leaves. The results showed that the fluorescence signals of the CiABI3-GFP and CiFUS3-GFP fusion proteins were both detected in the nucleus, indicating that the CiABI3 and CiFUS3 proteins are localized in the nucleus (Figure 9).

## 3. Discussion

The pecan (*Carya illinoinensis*) holds substantial economic value and positively impacts upon ecosystems and society [18]. Recently, researchers have made substantial progress in understanding pecans, particularly in seed development and lipid biosynthesis [16,19]. Comprehensive studies involving metabolomic and transcriptomic analyses have revealed significant metabolic changes and identified key transcription factors that are involved in lipid biosynthesis during pecan seed development. The observed dynamic changes in the metabolome highlight the complex regulatory mechanisms governing pecan seed maturation and lipid accumulation.

### 3.1. Lipid Metabolism and Fatty Acid Composition in Pecan Embryo During Seed Development

The untargeted metabolomic analysis showed significant changes in the metabolite profiles across different stages of seed development. The identification of 293 differentially accumulated metabolites (DAMs) highlights the complexity of metabolic adjustments during this process. The prominent presence of lipids and lipid-like molecules among the DAMs, comprising 33.11% of the total, highlights the central role of lipid biosynthesis in pecan seed development. This observation aligns with the previous studies that have reported substantial lipid accumulation in maturing seeds of other plant species, including *Arabidopsis* and oilseed plants like oil palm and soybean [20,21]. The identification of 97 DAMs related to lipids and lipid-like molecules highlights the critical role of lipid biosynthesis during pecan seed development (Figure 3). The predominance of glycerophospholipids and fatty acyls among these DAMs aligns with previous studies that highlight the importance of these lipid classes in seed maturation and oil accumulation [19,22]. The late-stage peak in DAM accumulation, particularly at S3, indicates a significant up-regulation of the lipid metabolic pathways as the seed matures [16].

The continuous increase in most FA components from S1 to S3, particularly the dramatic increases in palmitic acid, stearic acid, and oleic acid, reflects the developmental shifts in lipid storage within the embryo (Table 1). Similar trends have been observed in other oilseed species, such as walnuts, where specific FAs accumulate predominantly during the later stages of seed development [23]. The rapid accumulation of oleic acid during the early mature stage suggests its crucial role in establishing the oil content and quality of pecan seeds [2]. Oleic acid and linoleic acid were the most abundant unsaturated FA components in pecan seeds, which played important roles in human nutrition and health [24,25].

### 3.2. Transcriptional Regulation and Key Transcription Factor Analysis During Pecan Seed Development

The transcriptome provides all gene-expression data at the RNA level, and the transcriptomic analysis provides an investigation into the molecular regulation of pecan seed development. Plant lipid biosynthesis is connected to seed development, which is regulated by a set of key TF genes. The transcriptome analysis revealed a substantial number of differentially expressed genes, including 1201 transcription factors, which were classified into 65 families (Figure 6). The predominance of ERF, MYB, bHLH, C2H2, NAC, WRKY, and bZIP families among the DEGs highlights their pivotal roles in regulating seed development and lipid biosynthesis [1]. Previous studies have demonstrated the involvement of these TF families in various plant metabolic processes, including the abiotic stress responses in the pecan [26].

Comparative analyses with other species reveal conserved and unique aspects of lipid biosynthesis in the pecan. For instance, the roles of WRKY and bZIP TFs in lipid metabolism have been well-documented in the oil palm and the soybean, suggesting conserved regulatory mechanisms [27,28]. However, the distinct expression patterns of certain TFs in the pecan highlight species-specific regulatory networks that permit further investigation.

The qRT–PCR validation of selected TF genes corroborated the RNA-Seq data, affirming the reliability of our results. The significant up-regulation of genes, such as *CiABI3* and *CiFUS3,* during seed development emphasizes their regulatory roles in lipid biosynthesis, which is consistent with their known functions in other species [29,30]. As demonstrated through GFP fusion experiments, the nuclear localization of CiABI3 and CiFUS3 proteins further supports their involvement in transcriptional regulation within the nucleus. This nuclear localization pattern is also consistent with previous studies on ABI3 and FUS3 in *Arabidopsis*, and the two TFs have been shown to regulate key aspects of seed development and oil accumulation [31]. WRINKLED1 (WRI1) is a core TF, which belongs to the APETALA2/ETHYLENE RESPONSIVE FACTOR gene family. WRI1 controls the expression of a set of gene-encoding enzymes involved in lipid biosynthesis, such as ACCase, MAT, KASIII, KASI, and EAR [32]. In a previous study, *CiWRI1* was significantly induced at DAF131 and DAF138 in two pecan cultivars. Overexpression of *CiWRI1* in *Arabidopsis wri1-1* mutant restored lipid synthesis, and further analysis indicated that *CiWRI1* regulated oil synthesis by controlling *CiBCCP2* expression [33]. During plant oil biosynthesis, ABI3 interacts with FUS3 and WRI3 to increase carbon flux toward FA biosynthesis and enhance TAG production [34].

Our integrated metabolome and transcriptome analysis provide a detailed view of the molecular changes during pecan seed development. The identification of key TFs and their expression patterns provides insights into the regulatory networks controlling lipid biosynthesis. Validation of these findings through qRT–PCR and subcellular localization studies further strengthens our understanding of the roles these TFs play in seed development. These results enhance our knowledge of the molecular mechanisms governing pecan seed development and pave the way for future functional studies. By targeting specific TFs and regulatory networks, it may be possible to manipulate lipid content and improve seed quality in the pecan and other crops.

## 4. Materials and Methods

### 4.1. Plant Materials

Healthy nine-year-old pecan trees were cultivated in an orchard located in Jurong City, China (31°52′47″ N, 119°9′5″ E). The orchard experienced an average annual precipitation of 1018.6 mm and a mean annual temperature of 15.6 °C. Pecan fruits from the commercial pecan cultivar ‘Pawnee’ (the sequenced genotype) were harvested at three different developmental stages, namely at 120 (stage 1, S1), 135 (stage 2, S2), and 150 (stage 3, S3) days after flowering, between August and October. After removing the pericarp and seed coat, the embryo samples were quickly frozen in liquid nitrogen and stored at −80 °C for further experiments. Each sample was obtained from at least ten pecan fruits, and three biological replicates were used for each time point.

### 4.2. Oil Content and Fatty Acid Component Determination

The embryo samples from the pecan cultivar ‘Pawnee’ were harvested at three different developmental stages for oil content and fatty acid component analysis. The oil content of the embryos from three stages was determined via the Soxhlet method, as described previously [35].

The fatty acid components were analyzed using gas chromatography–mass spectrometry (Agilent GC–MS 7890A, Agilent Technologies, Santa Clara, CA, USA), as previously described [18]. The content of each fatty acid component was calculated using the peak area normalization method based on the GC–MS results.

### 4.3. Metabolomics Analysis

For untargeted metabolomics analysis, embryo samples from the three stages (S1, S2, and S3) collected previously were used. The samples were placed in an EP tube, and an extraction solution (acetonitrile: methanol = 1:1, containing an isotopically labeled internal standard mixture including ribitol) was added [36]. The samples were then vortexed for 30 s. They were then sonicated for 10 min in an ice-water bath and incubated for 1 h at −40 °C to precipitate proteins. After incubation, the sample was centrifuged at 12,000 rpm for 15 min. The resulting supernatant was transferred into a 2 mL LC/MS glass vial for UHPLC-QE-MS analysis. A quality control (QC) sample was prepared by combining an equal amount of the supernatants from all embryo samples.

The liquid chromatography–tandem mass spectrometry (LC-MS/MS) analyses were carried out using a UHPLC system (Vanquish, Thermo Fisher Scientific, Waltham, MA, USA) equipped with a UPLC BEH Amide column connected to a Q Exactive HFX mass spectrometer (Orbitrap MS, Thermo). The QE HFX mass spectrometer operated in information-dependent acquisition (IDA) mode controlled by the Xcalibur software version 4.1 (Thermo Fisher Scientific, Waltham, MA, USA) to assess the full-scan MS spectrum. The metabolomics analysis was conducted by Genedenovo Biotechnology Co., Ltd. (Guangzhou, China).

The raw data were converted to the mzXML format using ProteoWizard version 3.0.22167 and then processed using R language version 4.0.3 with XCMS. Metabolite annotation was conducted using an in-house MS2 database (BiotreeDB). Metabolites with a *t*-test *p* < 0.05 and variable importance in projection (VIP) ≥ 1 were considered as differentially accumulated metabolites (DAMs) between the two samples.

The KEGG (Kyoto Encyclopedia of Genes and Genomes) enrichment analyses of DAMs were investigated via Metaboanalyst 5.0.

### 4.4. RNA Extraction and Quality Assessment

Total RNA was extracted from pecan embryo samples using the TRIzol reagent (Invitrogen, Carlsbad, CA, USA) following the manufacturer’s instructions. Subsequently, the extracted RNA samples were treated with RNase-free DNase I (Qiagen, Hilden, Germany) to eliminate contaminating genomic DNA. The RNA concentration was measured using a NanoDrop 2000 spectrophotometer (Thermo Scientific, Wilmington, NC, USA), and the RNA integrity was assessed using the Agilent Bioanalyzer 2100 system (Agilent, Santa Clara, CA, USA).

### 4.5. Transcriptomics Analysis

The mRNA was enriched using oligo(dT) beads. The enriched mRNA was then fragmented into short sequences using the NEBNext Ultra RNA Library Prep Kit for Illumina (NEB #7530, New England Biolabs, Ipswich, MA, USA). Subsequently, the fragmented mRNA was reverse-transcribed to synthesize cDNA. The purified double-stranded cDNA fragments were subjected to end repair and A-tailing, followed by the ligation of Illumina sequencing adapters. After the ligation reaction, the products were purified using AMPure XP beads (1.0x volume) and then reverse-transcribed to construct the cDNA library.

After ensuring the quality of the cDNA library, it was sequenced on the Illumina Novaseq 6000 platform (Genedenovo Biotechnology Co., Guangzhou, China). To obtain high-quality sequencing reads, the raw sequences were filtered using FASTP software (v0.20.0). After quality control, the sequences were mapped to the reference genome using HISAT software (version 2.0.4) [37,38]. Subsequently, the aligned sequences were then assembled using StringTie software (v1.3.1) and quantified for gene-expression levels using the RSEM software v1.2.8 (http://deweylab.github.io/RSEM/ (accessed on 18 March 2024)) to calculate the fragments per kilobase of transcript per million mapped reads (FPKM) [39,40].

The identification of differentially expressed genes (DEGs) was carried out using the DESeq2 software (version 1.22.1). Genes with a log2(fold change) ≤ −1 or ≥1 and a false discovery rate (FDR) of less than 0.05 were considered significant. Subsequently, the expression trends of these DEGs were categorized using the Short Time-series Expression Miner (STEM) tool (http://www.cs.cmu.edu/~jernst/stem/ (accessed on 18 March 2024)) [41].

The KEGG enrichment analyses of the DEGs were performed to investigate the roles of DEGs in biological functions.

### 4.6. Identification of Differentially Expressed Transcription Factors

Transcription factors among the differentially expressed genes were identified and classified using the online tool ITAK (http://itak.feilab.net/cgi-bin/itak/index.cgi (accessed on 18 March 2024)) [42]. The transcription factor genes with FPKM values less than 5.0 at all time points were excluded from further analysis [18].

### 4.7. Validation of the Expression Patterns of Genes

In order to analyze the candidate TF genes using qRT–PCR (quantitative real-time PCR), 1 µg of total RNA was used to synthesize first-strand cDNA with the Prime-Script RT reagent kit (Takara, Dalian, China). Subsequently, the qRT–PCR experiments were conducted on an ABI 7500 Real-Time PCR System (Applied Biosystems™, Foster City, CA, USA) using the TB Green™ Premix Ex Taq™ II (Takara, Dalian, China). The specific primers for the candidate genes were designed using the IDT PrimerQuest online tool (https://sg.idtdna.com/PrimerQuest/Home/Index (accessed on 18 March 2024)). The Actin gene (*CiPaw.03G124400*), previously employed as a reference gene [43], was used for normalization. The relative expression levels of the genes were calculated using the 2^−∆∆Ct^ method [44]. The PCR cycling conditions were as follows: an initial denaturation at 95 °C for 30 s, followed by 40 cycles of 95 °C for 5 s and 60 °C for 15 s.

### 4.8. Correlation Analysis

Pearson correlation coefficients (PCCs) of the gene pairs were calculated by SPSS Statistics 25 (SPSS, Inc., Chicago, IL, USA) based on their expression data. The gene pairs whose PCC value was greater than 0.9 (*p* < 0.05) were selected and visualized with Cytoscape software (version 3.10.2).

### 4.9. Subcellular Localization Analysis

To analyze the localization of the TF genes, the coding regions of *CiABI3* and *CiFUS3* were amplified by PCR. Then, the PCR products were cloned upstream of the green fluorescent protein (GFP) gene into the pBWA(V)HS vector to generate the *CiABI3*-GFP and *CiFUS3*-GFP constructs, which were driven by the CaMV 35S promoter. Subsequently, the recombinant constructs were then transformed into tobacco leaves using Agrobacterium-mediated transformation. After two days of co-cultivation in the dark, fluorescence signals were detected and photographed using a C2-ER confocal microscope (Nikon, Kyoto, Japan). The empty pBWA(V)H-GLosgfp vector was used as a control.

### 4.10. Statistical Analysis

Gene-expression data during pecan seed development were obtained from at least three independent biological replicates and presented as mean values with standard errors (SE). To analyze significant differences at various stages, a one-way ANOVA followed by Duncan’s multiple range test (*p* < 0.05) was conducted using SPSS Statistics v25 (SPSS, Inc., Chicago, IL, USA).

## Figures and Tables

**Table 1:** Fatty acid composition in developing pecan embryo samples (μg/g). Different letters in a row indicate significant difference (*p* < 0.05) according to Duncan’s multiple range test. ND: not detected.

Fatty Acid Composition		Stages (Mean ± SD)		
		S1	S2	S3
Saturated FA	myristic acid (C14:0)	21.32 ± 0.2 c	58.60 ± 1.79 b	89.88 ± 6.89 a
	pentadecanoic acid (C15:0)	18.71 ± 0.13 b	29.94 ± 0.70 a	31.20 ± 2.11 a
	palmitic acid (C16:0)	1644.56 ± 52.69 c	6943.99 ± 141.99 b	9363.03 ± 388.72 a
	margaric acid (C17:0)	18.34 ± 0.22 c	88.08 ± 1.66 b	151.94 ± 7.89 a
	stearic acid (C18:0)	503.17 ± 12.17 c	2806.48 ± 42.13 b	5597.37 ± 406.79 a
	tricosylic acid(C23:0)	33.23 ± 0.71 b	36.93 ± 1.78 a	36.20 ± 1.22 a
	lignoceric acid (C24:0)	6.08 ± 0.23 c	12.76 ± 0.46 b	18.49 ± 0.53 a
Unsaturated FA	palmitoleic acid (C16:1)	29.73 ± 0.36 c	148.39 ± 1.21 b	180.95 ± 8.95 a
	heptadecenoic acid (C17:1)	ND	76.31 ± 1.08 b	109.99 ± 4.01 a
	oleic acid (C18:1)	7058.15 ± 112.49 c	74,071.92 ± 3643.51 b	105,881.08 ± 7862.46 a
	eicosenoic acid (C20:1)	41.72 ± 0.97 c	162.06 ± 2.65 b	298.29 ± 25.06 a
	nervonic acid (C24:1)	8.50 ± 0.15 c	14.37 ± 0.57 b	23.37 ± 1.69 a
	linoleic acid (C18:2n6)	5721.37 ± 84.07 c	28,959.31 ± 683.77 b	43,588.83 ± 3139.27 a
	α-linolenic acid (C18:3n3)	43.75 ± 1.97 c	349.59 ± 8.09 b	660.59 ± 47.86 a
	γ-linolenic acid (C18:3n6)	566.08 ± 6.26 c	1673.86 ± 19.92 b	2409.61 ± 200.06 a
	eicosatrienoic acid (C20:3n6)	6.41 ± 0.06 c	14.69 ± 0.22 b	16.91 ± 0.46 a
	arachidonic acid (C20:4n6)	27.07 ± 0.13 c	47.41 ± 1.43 b	72.88 ± 4.02 a

## Data Availability

Data are contained within the article and the Supplementary Materials. Raw transcriptomic data are available through NCBI BioProject (PRJNA1144794).

## Supplementary Materials

The following supporting information can be downloaded at https://www.mdpi.com/article/10.3390/ijms252111571/s1.
