# Supplementary material for: Metabolome and Transcriptome Analyses Reveal Metabolomic Variations and Key Transcription Factors Involved in Lipid Biosynthesis During Seed Development in Carya illinoinensis
Source: Int J Mol Sci. 2024 Oct 28;25(21):11571. doi: 10.3390/ijms252111571 (PMC11546405; doi:10.3390/ijms252111571)
Supplement: Supplementary file 1 [file ijms-25-11571-s001.zip › Supplementary Figures.pdf]

Figure S1

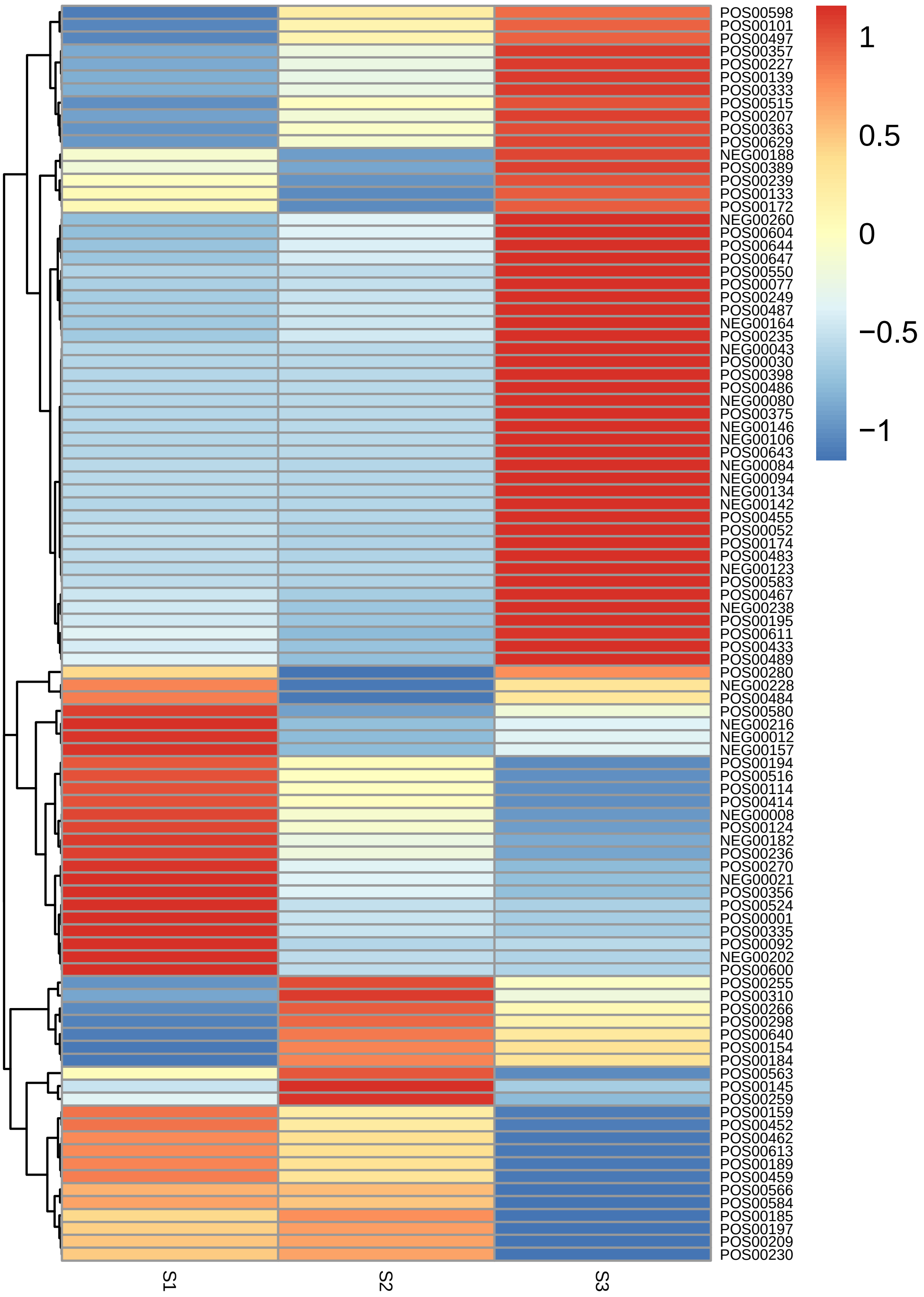

Figure S1 Expression of DAMs in the lipids and lipid-like molecules group.

Figure S2

S1 VS S2

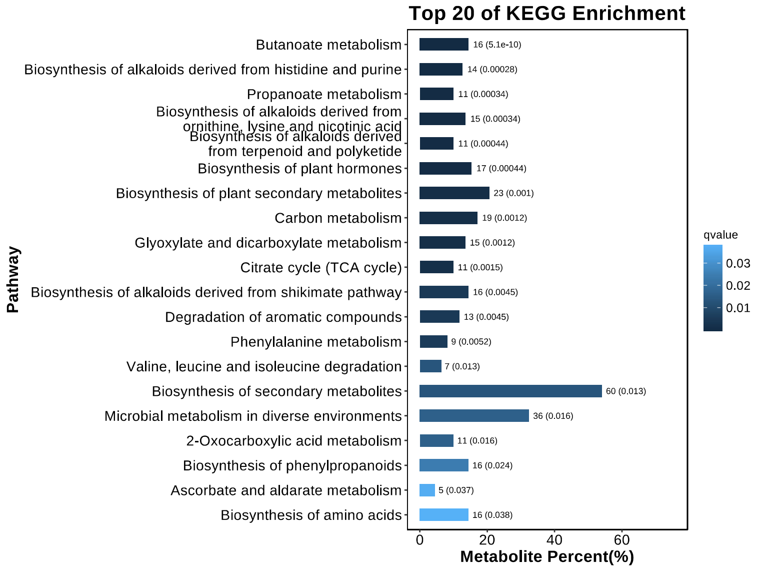

S1 VS S3

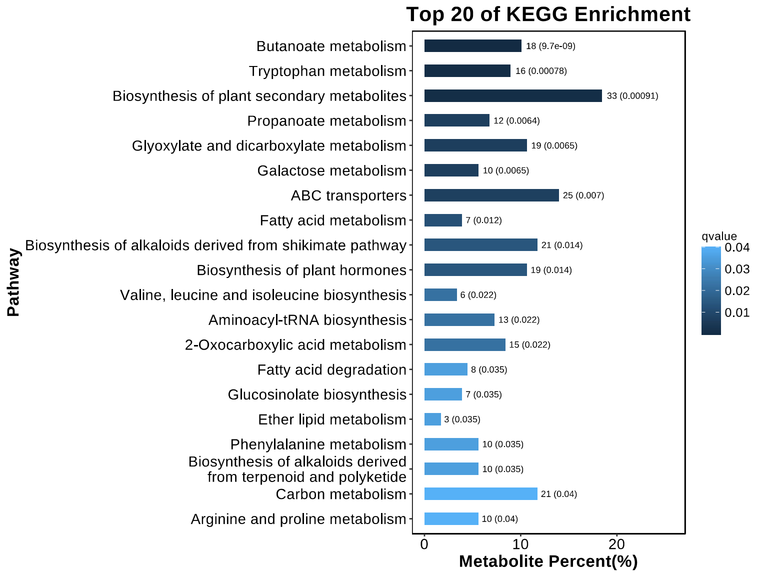

S2 VS S3

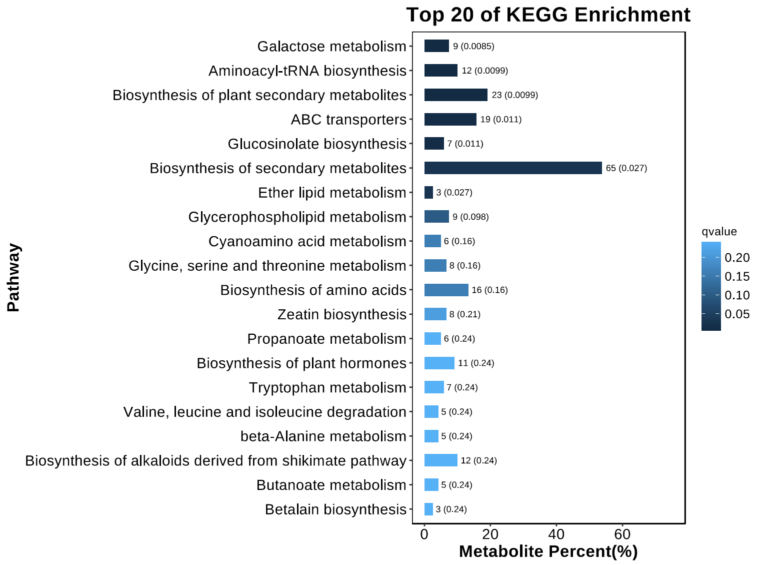

Figure S2 KEGG enrichment analysis of DAMs in three seed development stages of pecan.

Figure S3

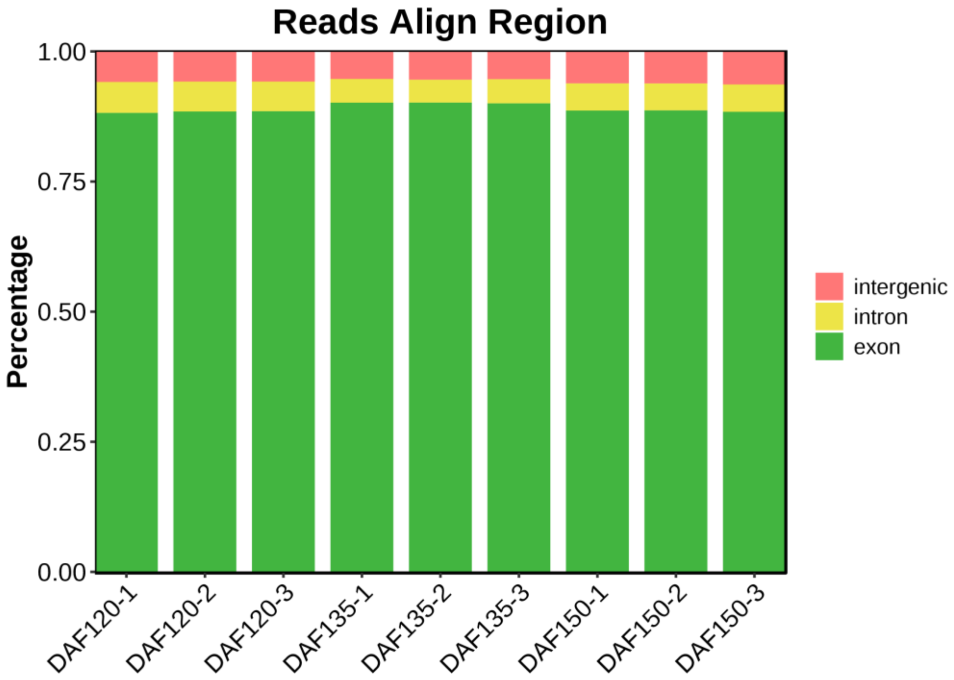

Figure S3 Distribution of reads in different regions of pecan genome.

Figure S4

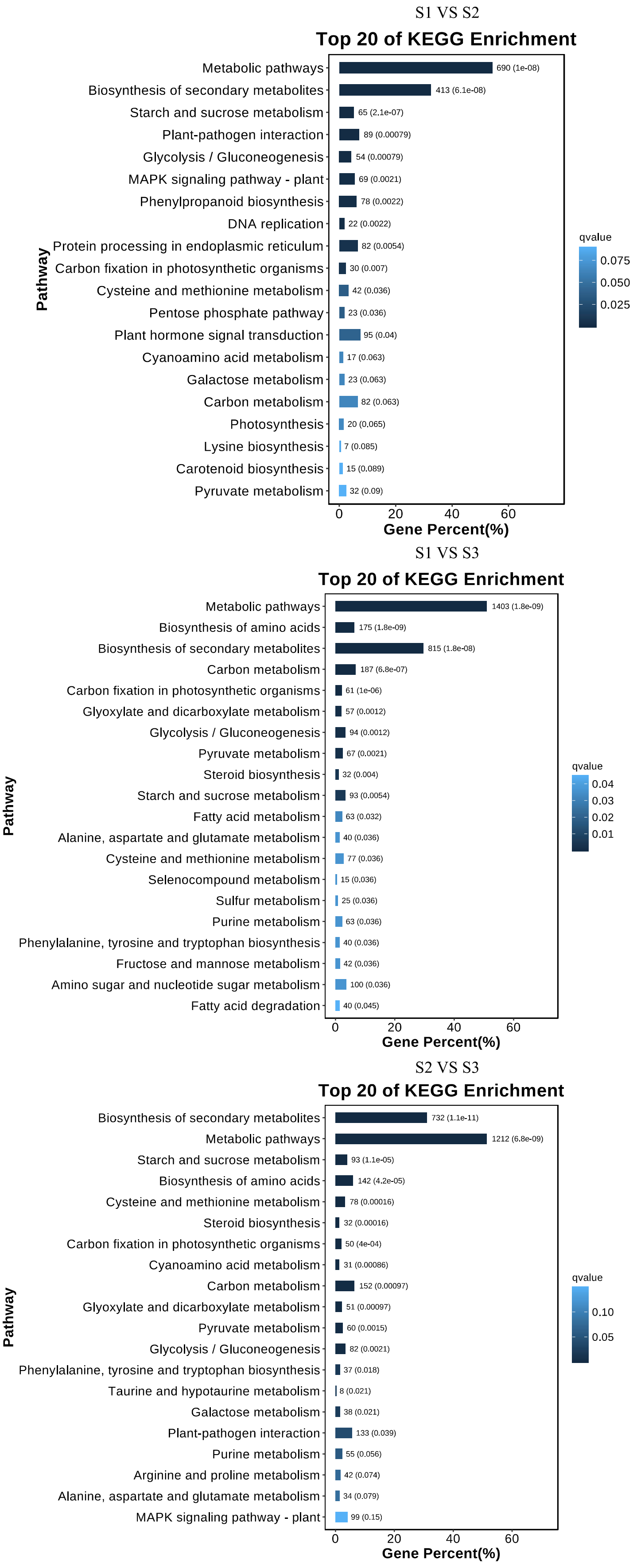
S1 VS S2
Top 20 of KEGG Enrichment
Metabolic pathways
Biosynthesis of secondary metabolites
Starch and sucrose metabolism
Plant-pathogen interaction
Glycolysis / Gluconeogenesis
MAPK signaling pathway - plant
Phenylpropanoid biosynthesis
DNA replication
Protein processing in endoplasmic reticulum
Carbon fixation in photosynthetic organisms
Cysteine and methionine metabolism
Pentose phosphate pathway
Plant hormone signal transduction
Cyanoamino acid metabolism
Galactose metabolism
Carbon metabolism
Photosynthesis
Lysine biosynthesis
Carotenoid biosynthesis
Pyruvate metabolism
690 (1e-08)
413 (6.1e-08)
65 (2.1e-07)
89 (0.00079)
54 (0.00079)
69 (0.0021)
78 (0.0022)
22 (0.0022)
82 (0.0054)
30 (0.007)
42 (0.036)
23 (0.036)
95 (0.04)
17 (0.063)
23 (0.063)
82 (0.063)
20 (0.065)
7 (0.085)
15 (0.089)
32 (0.09)
Pathway
qvalue
0.075
0.050
0.025
0
20
40
60
Gene Percent(%)
S1 VS S3
Top 20 of KEGG Enrichment
Metabolic pathways
Biosynthesis of amino acids
Biosynthesis of secondary metabolites
Carbon metabolism
Carbon fixation in photosynthetic organisms
Glyoxylate and dicarboxylate metabolism
Glycolysis / Gluconeogenesis
Pyruvate metabolism
Steroid biosynthesis
Starch and sucrose metabolism
Fatty acid metabolism
Alanine, aspartate and glutamate metabolism
Cysteine and methionine metabolism
Selenocompound metabolism
Sulfur metabolism
Purine metabolism
Phenylalanine, tyrosine and tryptophan biosynthesis
Fructose and mannose metabolism
Amino sugar and nucleotide sugar metabolism
Fatty acid degradation
1403 (1.8e-09)
175 (1.8e-09)
815 (1.8e-08)
187 (6.8e-07)
61 (1e-06)
57 (0.0012)
94 (0.0012)
67 (0.0021)
32 (0.004)
93 (0.0054)
63 (0.032)
40 (0.036)
77 (0.036)
15 (0.036)
25 (0.036)
63 (0.036)
40 (0.036)
42 (0.036)
100 (0.036)
40 (0.045)
Pathway
qvalue
0.04
0.03
0.02
0.01
0
20
40
60
Gene Percent(%)
S2 VS S3
Top 20 of KEGG Enrichment
Biosynthesis of secondary metabolites
Metabolic pathways
Starch and sucrose metabolism
Biosynthesis of amino acids
Cysteine and methionine metabolism
Steroid biosynthesis
Carbon fixation in photosynthetic organisms
Cyanoamino acid metabolism
Carbon metabolism
Glyoxylate and dicarboxylate metabolism
Pyruvate metabolism
Glycolysis / Gluconeogenesis
Phenylalanine, tyrosine and tryptophan biosynthesis
Taurine and hypotaurine metabolism
Galactose metabolism
Plant-pathogen interaction
Purine metabolism
Arginine and proline metabolism
Alanine, aspartate and glutamate metabolism
MAPK signaling pathway - plant
732 (1.1e-11)
1212 (6.8e-09)
93 (1.1e-05)
142 (4.2e-05)
78 (0.00016)
32 (0.00016)
50 (4e-04)
31 (0.00086)
152 (0.00097)
51 (0.00097)
60 (0.0015)
82 (0.0021)
37 (0.018)
8 (0.021)
38 (0.021)
133 (0.039)
55 (0.056)
42 (0.074)
34 (0.079)
99 (0.15)
Pathway
qvalue
0.10
0.05
0
20
40
60
Gene Percent(%)

Figure S4 KEGG enrichment analysis of DEGs in three seed development stages of pecan.

Figure S5

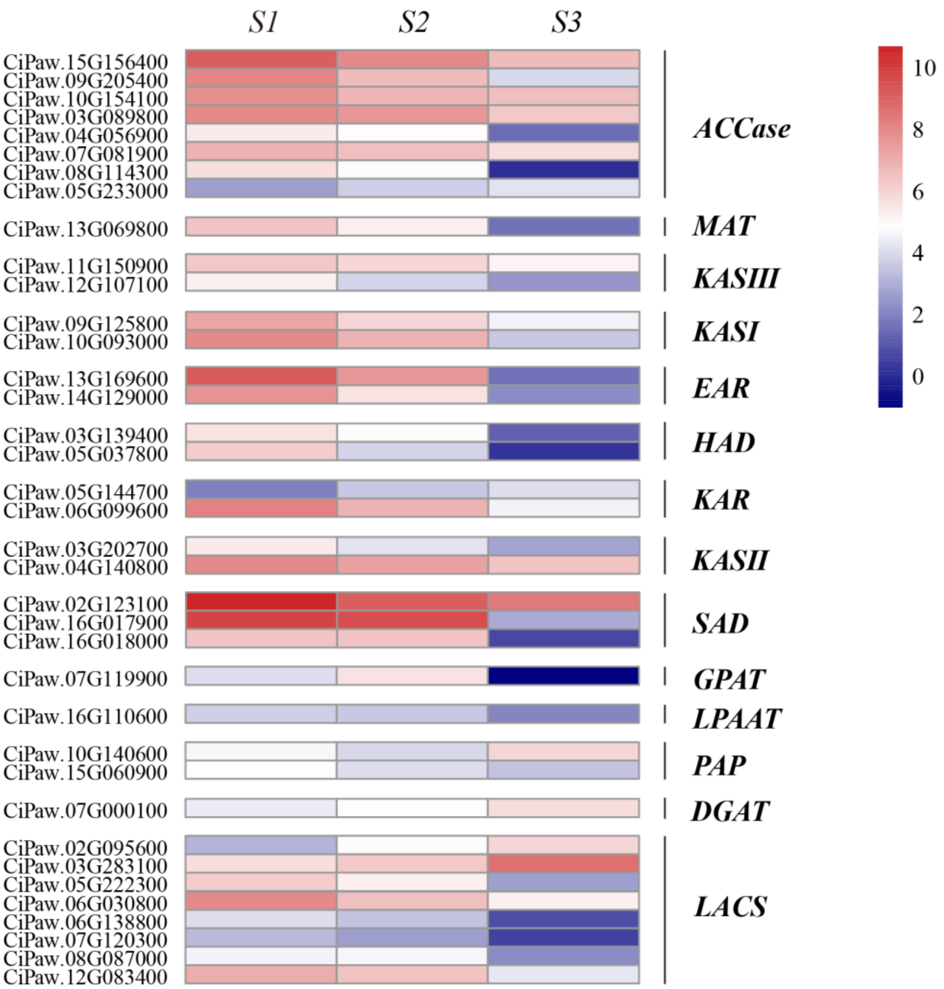

Figure S5 Expression of key genes involved in pecan seed development.

Figure S6

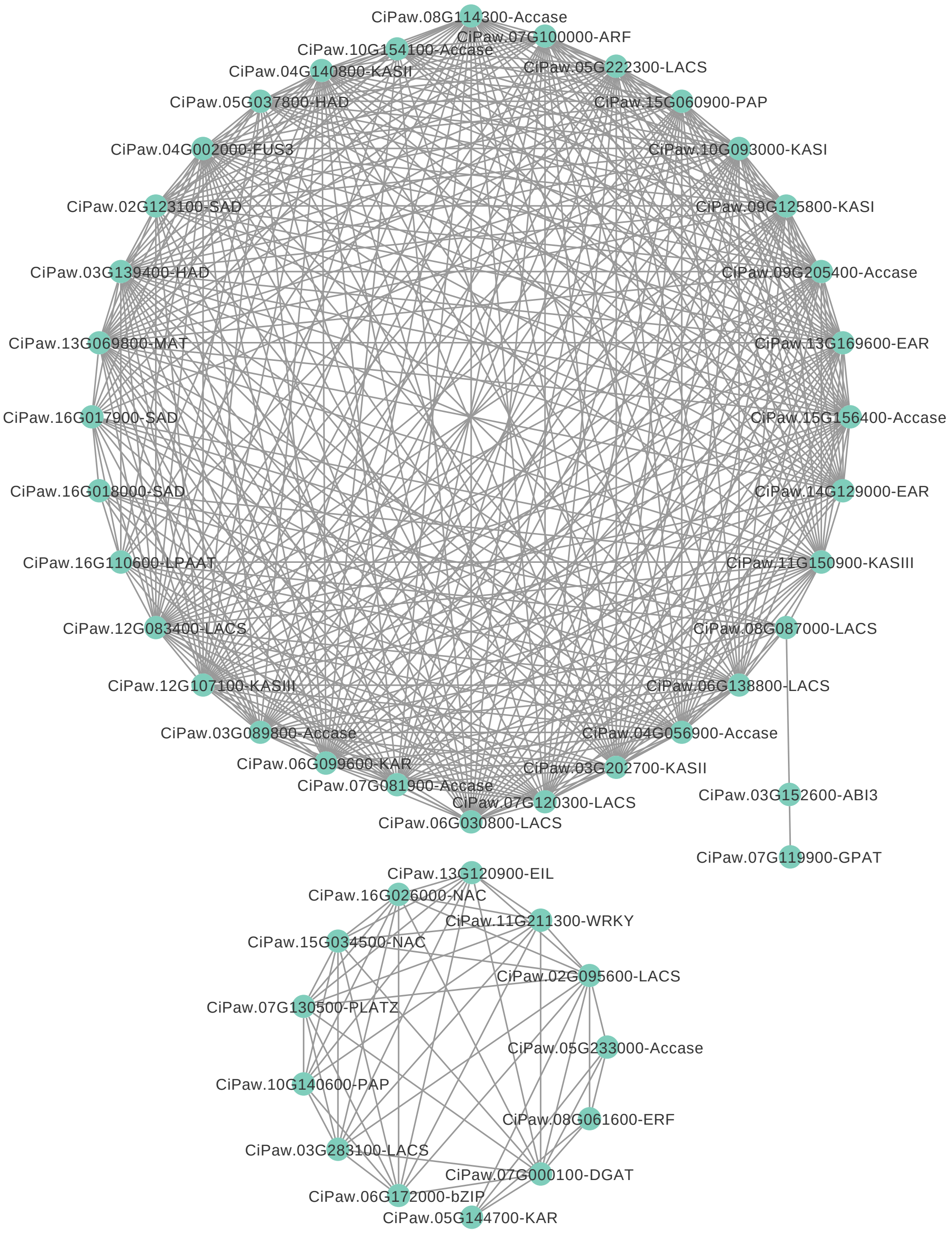

Figure S6 Co-expression networks of 10 key TF genes and 37 fatty acid metabolism related genes involved in pecan seed development.
